# Quality Improvement Project on Benzodiazepine Prescribing on Discharge and Compliance with Trust Guidelines

**DOI:** 10.1192/bjo.2026.11418

**Published:** 2026-06-30

**Authors:** Madeline Lewis, Daniel DiFrancesco, Lucas Jullian

**Affiliations:** Sussex Partnership trust, Eastbourne, United Kingdom

## Abstract

**Aims:**

Benzodiazepines are commonly prescribed for short-term treatment of anxiety andagitation on acute adult psychiatric wards, though long-term use can lead to dependence and withdrawal. ‘It is widely recognised that discharge prescriptions from mental health hospitals have high levels of prescribing and communication errors, and Sussex Partnership NHS Foundation Trust (SPFT) have written guidelines to ensure benzodiazepines are weaned effectively and communicated clearly to the GP.

- To improve compliance with Sussex Partnership Trust Guidelines.
- To provide safe weaning regimens for benzodiazepines.
- To provide clear handovers to GPs, and avoid unnecessary repeat prescriptions of benzodiazepines in the community.

**Methods:**

The standards audited against were the SPFT guidelines that 100% of patients should have:

1. The date of initiation and indication of benzodiazepine recorded.

No patient should be discharged from hospital on a benzodiazepine unless they were established on long-term treatment prior to admission or continued use is supported by a consultant psychiatrist.

If the benzodiazepine is expected to continue, the indication, duration, dose and details of any reducing regimen should be communicated to the GP.

A preliminary audit was completed for patients discharged from the Eastbourne Department of Psychiatry from September to October 2024. Data were taken from carenotes, discharge summary, scanned medication charts and ward review notes.

Following the preliminary audit, teaching on appropriate benzodiazepine prescribing was delivered to residents at the start of their rotation. Following this, we reaudited with same standards from October 2025 to January 2026.

**Results:**

In the initial audit, 12.5% were prescribed regular benzodiazepines and 15% PRN on discharge. Of these 80% patients were documented to be on long-term treatment and 40% had documented indication and reducing regimen.

In the repeat audit, 9% were prescribed regular benzodiazepines on discharge and 0% PRN on discharge. Of this 100% of patients had this supported by a consultant and had indication, duration of treatment and reducing regimen documented.

**Conclusion:**

The reaudit showed a reduced frequency of prescribing of benzodiazepines on discharge, and all had a reducing regimen documented, demonstrating significant improvement. Due to difficulty in arranging teaching for the whole cohort of residents, the teaching and repeat audit focused only on the male ward at the Department of Psychiatry. The wards may have different prescribing patterns which limits the generalisability of the results. Future plans involve arranging a teaching session at the induction of new residents, and re-audit to see if the improved compliance is maintained.

